# Off-Grid Direction of Arrival Estimation Based on Joint Spatial Sparsity for Distributed Sparse Linear Arrays

**DOI:** 10.3390/s141121981

**Published:** 2014-11-20

**Authors:** Yujie Liang, Rendong Ying, Zhenqi Lu, Peilin Liu

**Affiliations:** School of Electronic Information and Electrical Engineering, Shanghai Jiaotong University, 800 Dongchuan Road, Shanghai 200240, China; E-Mails: rdying@sjtu.edu.cn (R.Y.); zhenqilu2014@gmail.com (Z.L.); liupeilin@sjtu.edu.cn (P.L.)

**Keywords:** off-grid, joint spatial sparsity, distributed sparse linear arrays, direction of arrival estimation, concatenated atomic norm, semidefine program, distributed compressed sensing

## Abstract

In the design phase of sensor arrays during array signal processing, the estimation performance and system cost are largely determined by array aperture size. In this article, we address the problem of joint direction-of-arrival (DOA) estimation with distributed sparse linear arrays (SLAs) and propose an off-grid synchronous approach based on distributed compressed sensing to obtain larger array aperture. We focus on the complex source distribution in the practical applications and classify the sources into common and innovation parts according to whether a signal of source can impinge on all the SLAs or a specific one. For each SLA, we construct a corresponding virtual uniform linear array (ULA) to create the relationship of random linear map between the signals respectively observed by these two arrays. The signal ensembles including the common/innovation sources for different SLAs are abstracted as a joint spatial sparsity model. And we use the minimization of concatenated atomic norm via semidefinite programming to solve the problem of joint DOA estimation. Joint calculation of the signals observed by all the SLAs exploits their redundancy caused by the common sources and decreases the requirement of array size. The numerical results illustrate the advantages of the proposed approach.

## 1. Introduction

Direction of arrival (DOA) estimation using sensor arrays plays an important role in many applications of radar, sonar, ultrasonic, acoustic, and communication systems to track and localize sources [1–4]. Conventional DOA estimation methods obtain high freedoms by using arrays with large aperture. The increase in the demand of sensors enlarges the array aperture but also increases the cost of receiver hardware and computational complexity. Co-arrays based method is an effective way to enlarge the array aperture with no sensors added, which means the DOA information of the same sources can be estimated by fewer sensors. Second-order statistics is unitized in the covariance matrix of observed signals in the uniform linear array (ULA) to extend aperture, which achieves *O*(*N*) freedoms with *O*(*N*) sensors [5–7]. Four-order cumulants and higher-order statistics have been also used to make the array aperture larger [8,9]. The redundancy among the signals of common sources observed by every sensor in different arrays is considered and two or more arrays of specific structure are combined to construct a new virtual array with super large aperture [10–15]. MUSIC [16] and ESPRIT [17] were two famous algorithms to solve the problem of DOA estimation with high resolution. Some new array geometries except for ULA are proposed to further enhance this advantage. Minimization redundancy linear array (MRLA) [18] was used in the sensor array [10] and the multiple-input multiple-output (MIMO) radar [11] to obtain the larger array aperture. However, the implementation difficulty of MRLA blocks the development of this method. Pal and Vaidyanathan nested two or more ULAs and provided a nested array to solve the DOA estimations with high degrees of freedom, which is up to *O*(*N*^2^*^k^*) with 2*kN* sensors [12]. And then, they proposed a new approach for high resolution line spectrum estimation in both temporal and spatial domain using a co-prime pair of samplers [13]. Two uniform samplers with sample spacings *MT* and *NT* are used where *M* and *N* are co-prime and *T* has the dimension of space or time, which achieves *O*(*MN*) freedoms with *M* + *N* sensors. The implementations of these two array structures are easier than that of MRLA.

However, a more flexible structure of array like sparse linear array (SLA) is expected to estimate the DOA of sources together and developed by the revolution of compressed sensing (CS) [19]. To reduce the demand of sensors with no accuracy decreasing, a convex optimization method was used to deal with the problem of DOA estimation [20–23]. Although, *ℓ*_0_-norm of the incident signals is a rigorous way to model the sparsity constraint on the spatial distribution of sources, the *ℓ*_0_-norm-based optimization problem is not convex, and it is NP-hard to solve it directly [24]. Thus, convex relaxation is used to transform this problem into a convex one [22] and *ℓ*_1_-norm is introduced to solve it via a convex optimization process [20,21,25,26]. The recent development of CS theory [19] has verified the effectiveness of the application of sparse recovery technique with convex optimization [27] to DOA estimation with the same array structure. A sparse representation of signal in the space domain and its corresponding covariance vectors are comprehensively studied in [28,29]. And, Liu and Sha further extended their own wideband covariance matrix sparse representation method to focus on DOA estimation of wideband signals, respectively [30,31]. However, these CS-based methods are all constrained to study the sources sparse distributed on a pre-defined grid. Tan *et al.*, used joint sparsity reconstruction methods based on co-prime array to explore the underlying structure between sparse signals and gird mismatch [14]. Based on the developing theory of super resolution, they utilized the degrees of freedom for the co-prime arrays and proposed a sparse recovery method via total variation to obtain higher resolution [15].

In the practical applications of multiple arrays (such as wireless array sensor network, partial discharge location [32], volcano monitoring [33] and underwater monitoring [34]), the localized sources for each array maybe different which makes the existing co-arrays based methods out of action. In this article, we focus on the problem of joint DOA estimations for multiple distributed SLAs with the coexistence of common and innovation sources. The sources are classified according to whether they are observed by all the SLAs (The source observed by all the arrays named the common source and that observed by a specific array named the innovation source). This situation has not been discussed in the existing off-grid methods with co-arrays [15]. We can only obtain these DOA information via the accurate estimation algorithms separately implemented in different arrays. No advantage of joint calculation appears by using the process of centralized processing. But the observed signals of common sources in different arrays have redundancy which brings about repeated calculations in the DOA estimations. In our prior work, a joint frequency sparsity (JFS) model was built up and the distributed compressed sensing (DCS) off the grid was studied [35,36]. We introduce this idea of off-grid DCS into solving the problem of joint DOA estimation with distributed SLAs under the coexistence of common/innovation sources. An off-grid synchronous approach is proposed to utilize the observed signals in different arrays to estimate the DOA information together. We construct a virtual-ULA (VULA) for each SLA and consider the observed signals in a SLA as the random measurements of the observed signal in its corresponding VULA. The relationship between these two signals can be described as a random linear map. As assumed that the observed signal ensemble of each SLA is composed of sparsely distributed common and innovation sources, each ensemble is abstracted as a joint spatial sparsity (JSS) model under continuous atomic basis and described by a concatenated atomic norm (CA-norm). Since the compressed linear relation, the problem of joint DOA estimation is reformulated as the minimization of CA-norm. Semidefine program (SDP) is used to search the accurate solution under the condition of under-sampling. By exploiting the continuous counterpart [37] of the JSS model, off grid estimation results is achieved with no decreasing of freedom. Also, joint calculation exploits the redundancy among the signals of common sources observed by different SLAs to decrease array aperture size. Numerical results are given to illustrate the effectiveness of our approach and its advantage over the conventional methods of separate DOA estimation, which indicate a significant reduction in array aperture with the same estimation accuracy.

In this article, we use capital italic bold letters to represent matrices, and lowercase italic bold letters to represent vectors. For a given matrix *A*, A* denotes the conjugate transpose of *A*, *A^T^* denotes the regular transpose, and *A^H^* denotes the conjugate without transpose. For a given *p*, ‖*p*‖_0_, ‖*p*‖_1_ are the *ℓ*_0_ and *ℓ*_1_ norms, respectively, *p_i_* to represent the *i*-th element in *p*. ‖·‖_
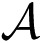
_ is the atomic norm and ${\left\|\cdot \right\|}_{A}^{*}$ is the dual norm of it. We use ⊗) to denote the Kronecker product of two matrices, and ⊙ to denote the point-wise multiplication of two vectors with the same dimension.

## 2. System Model

### 2.1. Overview of System Model

In the problem of joint DOA estimation with distributed SLAs under the coexistence of common and innovation sources, the utilization of signal redundancy of common sources is the key. Simple combination of the observed signals in SLAs has no help for the existing SDP solution, though it has high resolution [14]. Our proposed joint estimation approach can be divided into three phases, including source classification, VLUA construction and random mapping.

In the phase of source classification, we implement the source classification and study the different impacts of common and innovation sources on the observed signals. For simplification, we just discuss the situation of the same sensor number and source number. A SLA can be seen as the random sample measurements of a ULA with the same array aperture, so that we construct a VLUA with the same structure corresponding to each SLA and build up a JSS model for the sparse spatial distributed common and innovation sources with this VLUA. A continuous atomic basis is used to give a more accurate solution. As the relationship of the signals observed by SLA and its corresponding VLUA is described by a random linear map, the problem of joint DOA estimation is similar to the problem faced with in DSC. We combine the observed signals into one and form a new joint DOA estimation problem. Because the factors of common sources in JSS models with different VLUAs are the same, they can be independent from the original observed signals and seen as one item in the joint estimation problem. Therefore, we can choose a norm minimization to indicate the problem and solve it via the optimization method.

### 2.2. A Multi-SLAs System with Common/Innovation Sources

**Figure 1.**
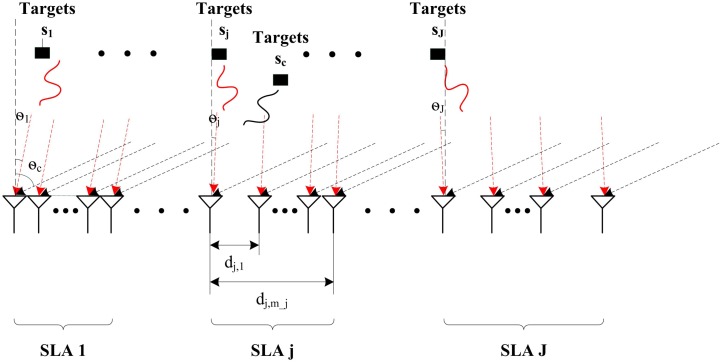
A practical multi-SLAs system with partly common sources.

Figure 1 shows a practical application of the typical array network system. The narrowband signals of *K* sources, sparsely distributed in the space domain, with the same wavelength, say λ, impinge on several SLAs. The signals of some sources can impinge on all arrays; but those of the others can only impinge on the specific ones, which are classified into the common sources and the innovation ones. We define the signal ensemble as the set of sources impinging on one array. There are *J* signal ensembles existing in the system. Let Λ = {1, 2, …, *J*} denote the set of indices for the *J* signal. The multi-SLAs system consists of *M* sensors with *J* SLAs placed according to the philosophy of MRLA, where each SLA contains *M_j_* = *M/J* sensors with the inter-element spacing being times of the half wavelength and the smallest inter-array spacing between the two consecutive SLA centers is larger than the largest size of SLA.

In the *j*-th ensemble, *K_c_* common sources and *K_j_* innovation sources simultaneously impinge on the *j*-th SLA. The common sources, of which the wave signals are denoted as *s_c_*,*_k_*(*t*), *k* = 1, …, *K_c_*, mean the sources in the ensemble impinge on all the SLAs. And the innovation sources, of which the wave signals are denoted as *s_c_*,*_j_*(*t*), *j* = 1, …, *K_j_*, *j* ∈ Λ, mean the sources in the ensemble impinge on only one SLA. Therefore, there are *K* = *K_c_* + Σ*_j_*_∈Λ_ *K_j_* sources in all to be localized in the system. Their powers are respectively

$${\left\{\sigma_{c,k}\right\}}_{k=1}^{K_{c}},{\left\{\sigma_{1,k}\right\}}_{k=1}^{K_{1}},\ldots ,{\left\{\sigma_{J,k}\right\}}_{k=1}^{K_{J}},$$

and the sources are sparsely distributed in the space domain with DOAs of

$${\left\{\theta_{c,k}\right\}}_{k=1}^{K_{c}},{\left\{\theta_{1,k}\right\}}_{k=1}^{K_{1}},\ldots ,{\left\{\theta_{J,k}\right\}}_{k=1}^{K_{J}}.$$

### 2.3. Joint Spatial Sparsity Model Based on the Virtual ULA

**Figure 2.**
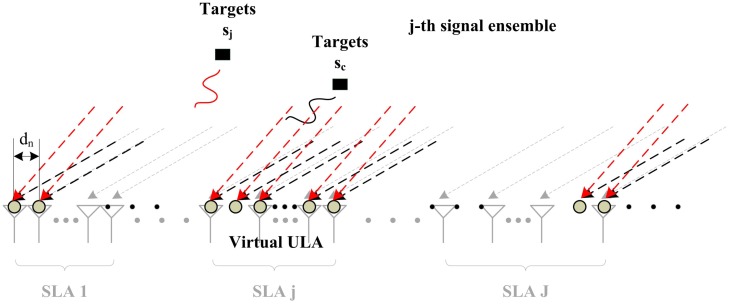
The structure of VULA for common/innovation sources.

For simplicity, we assume that all the signal sources in noiseless environment are far-field and temporally uncorrelated. To build up a complete JSS model, we first construct a VULA as a reference array with *N*(*N* ≫ *M_j_*) sensors shown in Figure 2, of which the inter-element spacing is the half wavelength. Each SLA in the system is regarded as *M_j_* random samplings of these *N* sensors. The first sensor of the first SLA in the system coincides with the first sensor in the VULA, defined as the reference element in the system. The array aperture of VULAs must be no smaller than the cumulative sum of the apertures of SLAs, *i.e.*,

$$\underset{n=1,\ldots ,N}{\max}\{d_{n}\}\geq \underset{m_{j}=1,\ldots ,M_{j},j\in \Lambda }{\max}\{d_{m_{j}}\}.$$

where, *d_n_* is the spacing between the *n*-th sensor and the reference element; *d_m_j__* is the spacing between the *m*-th sensor in the *j*-th SLA and the reference element.

Here, we give *J* signal ensembles corresponding to *J* SLAs in the system. Considering the signals of *K_c_* common sources and *K_j_* innovation sources simultaneously impinge on the *j*-th SLA in the practical system, we assume now they impinge on the VULA in the *j*-th signal ensemble. On the other hand, the sensors in the VULA in the *j*-th ensemble can sense the signals of *K_c_* + *K_j_* sources. Except for the signals ${\left\{s_{c,k}(t)\right\}}_{k=1}^{K_{c}}$ of the common sources, these sensors in one ensemble can only sense those ${\left\{s_{j,k}(t)\right\}}_{k=1}^{K_{j}}$ of the innovation ones. Thus, the signal observed by the *n*-th sensor in the *j*-th ensemble is expressed as

(1) $$x_{j,n}(t)=\sum_{k=1}^{K_{c}}s_{c,k}(t)e^{-j2\pi \frac{d_{n}}{\lambda }\sin(\theta_{c,k})}+\sum_{k=1}^{K_{j}}s_{j,k}(t)e^{-j2\pi \frac{d_{n}}{\lambda }\sin(\theta_{j,k})}$$

Since the inter-element spacing of the ULA is half length of the wave, *i.e.*, *d_n_* = (*n* − 1)λ/2, *n* = 1, …, *N*, Equation (1) is expressed as

(2) $$x_{j,n}(t)=\sum_{k=1}^{K_{c}}s_{c,k}(t)e^{-j\pi (n-1)\sin(\theta_{c,k})}+\sum_{k=1}^{K_{j}}s_{j,k}(t)e^{-j\pi (n-1)\sin(\theta_{j,k})}$$

The signals observed by the VULA with *N* sensors in the *j*-th ensemble have the vector form of

(3) $$x_{j}(t)=A_{c}s_{c}(t)+A_{j}s_{j}(t)$$

where, *s_c_* = [*s_c_*_,1_, …, *s_c_*,*_Kc_*]*^T^* is the common sources from *K_c_* different directions, and *s_j_* = [*s_j_*_,1_, …, *s_j_*,*_Kj_*]*^T^*, *j* ∈ Λ is the innovation sources from *K_j_* different directions. The matrix *A_c_* is the steering matrix of the first array for the common sources

(4) $$\begin{array}{ll} {\text{A}}_{c} & =[{\text{a}}_{c}(\theta_{c,1}),a_{c}(\theta_{c,2}),\ldots ,a_{c}(\theta_{c,K_{c}})]\\ & =\left[\begin{array}{cccc} 1 & 1 & \ldots & 1\\ e^{-j\pi \sin(\theta_{c,1})} & e^{-j\pi \lambda \sin(\theta_{c,2})} & \ldots & e^{-j\pi \lambda \sin(\theta_{c,K_{c}})}\\ \vdots & \vdots & \ddots & \vdots \\ e^{-j\pi (N-1)\sin(\theta_{c,1})} & e^{-j\pi (N-1)\sin(\theta_{c,2})} & \ldots & e^{-j\pi (N-1)\sin(\theta_{c,K_{c}})} \end{array}\right] \end{array}$$

where ***a**_c_*(*θ_c_*,*_k_*) = [1, *e*^−^*^jπ^*^(^*^n^*^−1)sin(^*^θc^*,*^k^*^)^, …, *e*^−^*^jπ^*^(^*^n^*^−1)sin(^*^θc^*,*^k^*^)^]*^T^*. The matrix ***A**_j_* is the steering matrix in the *j*-th ensemble for the innovation sources.

(5) $$\begin{array}{ll} A_{j} & =[a_{c}(\theta_{j,1}),a_{c}(\theta_{j,2}),\ldots ,a_{j}(\theta_{j,K_{j}})]\\ & =\left[\begin{array}{cccc} 1 & 1 & \ldots & 1\\ e^{-j\pi \sin(\theta_{j,1})} & e^{-j\pi \sin(\theta_{j,2})} & \ldots & e^{-j\pi \sin(\theta_{j,K_{j}})}\\ \vdots & \vdots & \ddots & \vdots \\ e^{-j\pi (N-1)\sin(\theta_{j,1})} & e^{-j\pi (N-1)\sin(\theta_{j,2})} & \ldots & e^{-j\pi (N-1)\sin(\theta_{j,K_{j}})} \end{array}\right] \end{array}$$

where ***a**_j_*(*θ_j_*_,k_) = [1, *e*^−^*^jπ^*^(^*^n^*^−1)sin(^*^θj^*,*^k^*^)^, …, *e*^−^*^jπ^*^(^*^n^*^−1)sin(^*^θj^*,*^k^*)]*^T^*.

As assumed that the sources are uncorrelated, the common source correlation matrix and the innovation source correlation matrix should be diagonal, namely, ***R**_ss_*,*_c_* = diag(*σ_c_*_,1_, …, *σ_c_*,*_k__c_*) and ***R**_ss_*,*_j_* = diag(*σ_j_*_,1_, …, *σ_j_*,*_Kj_*). Then the correlation matrix of the *j*-th ensemble is given by

(6) $$\begin{array}{ll} {\text{R}}_{xx,j} & =E[x_{j}(t)x_{j}(t)*]\\ & =A_{c}R_{ss,c}A_{c}^{*}+A_{j}R_{ss,j}A_{j}^{*}\\ & =\sum_{k=1}^{K_{c}}\sigma_{c,k}a_{c}(\theta_{c,k})a_{c}^{*}(\theta_{c,k})+\sum_{k=1}^{K_{j}}\sigma_{j,k}a_{j}(\theta_{c,k})a_{j}^{*}(\theta_{c,k}) \end{array}$$

After vectorizing the correlation matrix ***R**_xx_*,*_j_*, we have

(7) $$\upsilon_{j}=\text{vec}({\text{R}}_{xx,j})=\Phi_{c}(\theta_{c,1},\ldots ,\theta_{c,K_{c}})\sigma_{c}+\Phi_{j}(\theta_{j,1},\ldots ,\theta_{j,K_{j}})\sigma_{j}$$

where,

$$\begin{array}{ll} \Phi_{c}(\theta_{c,1},\ldots ,\theta_{c,K_{c}}) & =A_{c}^{*}⊙A_{c}\\ & =[a_{c}{\left(\theta_{1}\right)}^{H}⊗a_{c}(\theta_{1}),\ldots ,a_{c}{\left(\theta_{K_{c}}\right)}^{H}⊗a_{c}(\theta_{K_{c}})] \end{array}$$

$$\begin{array}{ll} \Phi_{j}(\theta_{j,1},\ldots ,\theta_{j,K_{j}}) & =A_{j}^{*}⊙A_{j}\\ & =[a_{j}{\left(\theta_{1}\right)}^{H}⊗a_{j}(\theta_{1}),\ldots ,a_{j}{\left(\theta_{K_{j}}\right)}^{H}⊗a_{j}(\theta_{K_{j}})] \end{array}$$

The signals of interests respectively become ***σ**_c_* = [*σ_c_*_,1_, …, *σ_c_*,*_k__c_*]*^T^* and ***σ**_j_* = [*σ_j_*_,1_, …, *σ_j_*,*_Kj_*]*^T^*.

As we extend the signals observed by the ULA in the *j*-th ensemble to those observed by the new array with *N*^2^ sensors, the signals observed by the *n*-th sensor in the new array is given by

(8) $$\upsilon_{j}(n)=\sum_{k=1}^{K_{c}}\sigma_{c}(k)e^{-j\pi (n-1)\sin(\theta_{c,k})}+\sum_{k=1}^{K_{j}}\sigma_{j}(k)e^{-j\pi (n-1)\sin(\theta_{j,k})},n=1,\ldots ,N^{2}$$

And the vector form is

(9) $$\upsilon_{j}=\left[\begin{array}{c} \sum_{k=1}^{K_{c}}\sigma_{c}(k)+\sum_{k=1}^{K_{j}}\sigma_{j}(k)\\ \vdots \\ \sum_{k=1}^{K_{c}}\sigma_{c}(k)e^{-j\pi \sin(\theta_{c,k})}+\sum_{k=1}^{K_{j}}\sigma_{j}(k)e^{-j\pi \sin(\theta_{j,k})}\\ \vdots \\ \sum_{k=1}^{K_{c}}\sigma_{c}(k)e^{-j\pi (N^{2}-1)\sin(\theta_{c,k})}+\sum_{k=1}^{K_{j}}\sigma_{j}(k)e^{-j\pi (N^{2}-1)\sin(\theta_{j,k})} \end{array}\right]$$

Actually, the vector *v_j_* has *N*^2^ elements but only 2*N* − 1 non-repeated values, which means the corresponding array has at most *N* − 1 degree of freedoms [12,13,16]. Thus, the SLA with *M_j_* ≪ *N* sensors can localize at most *O*(*N*) sources via CS [19,27].

With the consideration of the causality of system, we give a straightforward change of variables. Letting $\xi_{k}=\frac{1+\sin(\theta_{k})}{2}\in [0,1]$ for all *k*, the linear model of Equation (11) can be transformed into

(10) $$\begin{array}{ll} r_{j} & =e^{-j\pi n}\upsilon_{j}\\ & =\left[\begin{array}{c} \sum_{k=1}^{K_{c}}\sigma_{c}(k)+\sum_{k=1}^{K_{j}}\sigma_{j}(k)\\ \vdots \\ \sum_{k=1}^{K_{c}}\sigma_{c}(k)e^{-j2\pi \xi_{c,k}}+\sum_{k=1}^{K_{j}}\sigma_{j}(k)e^{-j2\pi \xi_{j,k}}\\ \vdots \\ \sum_{k=1}^{K_{c}}\sigma_{c}(k)e^{-j2\pi (N^{2}-1)\xi_{c,k}}+\sum_{k=1}^{K_{j}}\sigma_{j}(k)e^{-j2\pi (N^{2}-1)\xi_{j,k}} \end{array}\right] \end{array}$$

The sources in the system have JSS, which means each element of the vector ***r**_j_* is generated as a combination of two DOA sparse components (i) a common component *z_c_*, which is common for all the array ensembles; and (ii) an innovation component *z_j_*, which is unique to the corresponding ensemble. The vector ***r**_j_* can be expressed as

(11) $$r_{j}={\tilde{\Phi }}_{c}(\xi_{c,1},\ldots ,\xi_{c,Kc})\sigma_{c}+{\tilde{\Phi }}_{j}(\xi_{j,1},\ldots ,\xi_{j,Kj})\sigma_{j},j\in \Lambda$$

Due to the spatial sparsity of ***σ**_c_* and ***σ**_j_*, *r_j_* is a JSS setting. The components in each ensemble is denoted by $Z={\left[z_{c}^{*},z_{1}^{*},\ldots ,z_{J}^{*}\right]}^{*}$. These combine additively, giving *r_j_* = *z_c_* + *z_j_*,*j* ∈ Λ. And the two component can be expressed as

(12) $$\begin{array}{cc} z_{c}={\tilde{\Phi }}_{c}\sigma_{c} & =\left[\begin{array}{c} \sum_{k=1}^{K_{c}}\sigma_{c}(k)\\ \vdots \\ \sum_{k=1}^{K_{c}}\sigma_{c}(k)e^{-j2\pi \xi_{c,k}}\\ \vdots \\ \sum_{k=1}^{K_{c}}\sigma_{c}(k)e^{-j2\pi (N^{2}-1)\xi_{c,k}} \end{array}\right]\\ z_{j}={\tilde{\Phi }}_{j}\sigma_{j} & =\left[\begin{array}{c} \sum_{k=1}^{K_{j}}\sigma_{j}(k)\\ \vdots \\ \sum_{k=1}^{K_{j}}\sigma_{j}(k)e^{-j2\pi \xi_{j,k}}\\ \vdots \\ \sum_{k=1}^{K_{j}}\sigma_{j}(k)e^{-j2\pi (N^{2}-1)\xi_{j,k}} \end{array}\right] \end{array}$$

### 2.4. Random Linear Map from VULA to SLA

Actually, we attempt to design SLAs in the system by reducing the number of sensors equipped with their own corresponding VULA for low cost of system. *M_j_* sensors are active in every SLAs with the inter-element spacing of random times of the half wave length. And the number of sensors in each SLA is equal, *i.e.*, *M*_1_ = *M_j_* = *M/J*. Every SLA corresponding to the *j*-th ensemble has its *K_c_* common sources and *K_j_* innovation sources. We can get the signals in the ensemble collected by sensors the *j*-th SLA, *i.e.*,

(13) $$y_{j,m_{j}}(t)=\sum_{k=1}^{K_{c}}s_{c,k}(t)e^{-j2\pi \frac{d_{j,m_{j}}}{\lambda }\sin(\theta_{c,k})}+\sum_{k=1}^{K_{j}}s_{j,k}(t)e^{-j2\pi \frac{d_{j,m_{j}}}{\lambda }\sin(\theta_{j,k})}$$

and the vector form of which is given as

(14) $${\text{y}}_{j}(t)=\left[\begin{array}{c} \sum_{k=1}^{K_{c}}s_{c,k}(t)+\sum_{k=1}^{K_{j}}s_{j,k}(t)\\ \sum_{k=1}^{K_{c}}s_{c,k}(t)e^{-j2\pi \frac{d_{j,1}}{\lambda }\sin(\theta_{c,k})}+\sum_{k=1}^{K_{j}}s_{j,k}(t)e^{-j2\pi \frac{d_{j,1}}{\lambda }\sin(\theta_{j,k})}\\ \vdots \\ \sum_{k=1}^{K_{c}}s_{c,k}(t)e^{-j2\pi \frac{d_{j,M_{j}}}{\lambda }\sin(\theta_{c,k})}+\sum_{k=1}^{K_{j}}s_{j,k}(t)e^{-j2\pi \frac{d_{j,M_{j}}}{\lambda }\sin(\theta_{j,k})} \end{array}\right]\in {\mathbb{C}}^{M_{j}}$$

**Figure 3.**
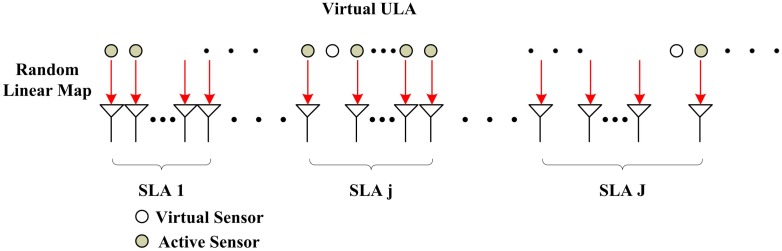
Random linear map for VULA to SLA.

Obviously, the data collected by a SLA can be considered as a random sampling of that sensed by its VULA shown in Figure 3, where some of sensors in ULA is not used in the practical SLAs, named virtual sensors. The relationship of linear map can be expressed as

(15) $${\text{y}}_{j}(t)=\Theta_{j}{\text{x}}_{j}(t)\in {\mathbb{C}}^{M_{j}}$$

where, different measurement matrix Θ*_j_* ∈ ℂ*^Mj×N^* corresponding to different choice of sensors is a random linear map from VULAs to SLAs. Obviously, Θ*_j_* is a random unit matrix. Therefore, the correlation matrix among the *j*-th ensemble can then expressed as

(16) $$\begin{array}{cc} R_{yy,j} & =E[y_{j}(t)y_{j}(t)*]\\ & =\Theta_{j}R_{xx,j}\Theta_{j}^{*} \end{array}$$

We spread the first row and the first column as a new vector according to the following rule

(17) $$u_{j}={\left[R_{yy,j}(0,M_{j}-1),\ldots ,R_{yy,j}(0,1),R_{yy,j}(0,0),R_{yy,j}(1,0),\ldots R_{yy,j}(M_{j}-1,0)\right]}^{T}$$

The vector ***u**_j_* ∈ ℂ^2^*^Mj^*^−1^ is also a random sampling of the vector *v_j_* in Equation (11) given as

(18) $$u_{j}=\Psi_{j}\upsilon_{j}$$

where, **ψ***_j_* ∈ ℂ^(2^*^M^^j^*^−1)^×*^N^*^2^ is a new random linear map. This matrix contains the *N* + 1 ± (*d_j_*_,1_ − *d_j_*,*_m_*,*_j_*)-th row (1 < *m_j_* ≤ *M_j_*) of a *N*^2^ × *N*^2^ unit matrix. By straightforward changing of variables, the linear model of Equation (18) is reformulated as

(19) $$w_{j}(\xi_{c,k},\xi_{j,k})=\Psi_{j}r_{j}(\xi_{c,k},\xi_{j,k})$$

Let $W={\left[w_{1}^{*},\ldots ,w_{J}^{*}\right]}^{*}\in {\mathbb{C}}^{2M-J},$ $R={\left[r_{1}^{*},\ldots ,r_{J}^{*}\right]}^{*}\in {\mathbb{C}}^{JN^{2}}$, and **ψ** = diag(**ψ**_1_, …, **ψ***_J_*) ∈ ℂ^(2^*^m^*^−^*^j^*^)×^*^JN^*^2^. The joint linear model of SLAs in the system is given as

(20) W=ΨR

## 3. SDP Based Joint DOA Estimation Algorithm

### 3.1. CA-Norm of JSS Model

The ill-posed inverse problem was studied by Chandrasekaran [38] and a general framework was provided to convert notions of simplicity into convex penalty functions, resulting in convex optimization solutions to linear undetermined inverse problems. Considering a simple model with a nonnegative combination of a few sensors from atomic set, signal *x* ∈ ℝ*^P^* can be formed as follows:

(21) $$x=\sum_{i=1}^{K}c_{i}a_{i},a_{i}\in A,c_{i}\geq 0$$

where 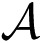 is a set of atoms that constitutes simple building blocks of general signals. And *x* is assumed simple so that *K* is relatively small. Then, the definition of atomic norm was given as [38]

(22) $${\left\|x\right\|}_{A}=\inf\left\{\sum_{a\in A}:x=\sum_{a\in A}c_{a}a,c_{a}\geq 0\forall a\in \Lambda \right\}$$

And the support function of 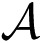 is given as:

(23) $${\left\|\text{x}\right\|}_{A}^{*}=\sup\{\langle x,a\rangle :a\in A\}$$

Equipped with a convex penalty function given a set of atoms, a convex optimization method can be used to recover a “simple” model given limited linear measurements. With a known linear map Φ : ℝ*^P^* → ℝ*^N^*, linear information about x* formed according to Equation (21) from a set of atoms is given as:

(24) $$y=\Phi \tilde{x}$$

The convex formulation to reconstruct ***x̃*** given ***y***:

(25) $$\begin{array}{ccc} \hat{x}=\arg\underset{x}{\min}{\left\|x\right\|}_{A} & s.t & y=\Phi x \end{array}$$

The dual problem of Equation (25) is given as follows:

(26) $$\begin{array}{ccc} \underset{z}{\max}y^{T}z & s.t. & {\left\|\Phi^{*}z\right\|}_{A}^{*}\leq 1 \end{array}$$

Obviously, Equation (20) is such an ill-posed inverse problem, when the number of available sensors of SLAs is smaller than the number of sensors in VULAs, *i.e.*, *M_j_* ≪ *N*. We can construct *J* + 1 atoms sets for the JSS model in Equation (11), respectively corresponding to the signal of common source *s_c_*(*t*) and the signal of innovation source *s_j_*(*t*), which take the same form of

(27) $$A=\{\begin{array}{cc} \alpha_{j}(\xi ,n):\xi \in [0,1], & n\in [0,1,\ldots ,N-1] \end{array}\}$$

where, *α*(*ξ*, *n*) = *e*^−^*^j^*^2^*^π^*^(^*^n^*^−1)^*^ξ^*. The sets of variables $\Omega_{c}={\left\{\xi_{c,k}\right\}}_{k=1}^{K_{c}}$ and $\Omega_{j}={\left\{\xi_{j,k}\right\}}_{k=1}^{K_{j}}$ can lie anywhere on the unite circle, such that *ξ_c_*,*_k_*, *ξ_j_*,*_k_* are continuously valued in [0,1].

In the prior work, we have considered a joint frequency sparsity (JFS) model and extended the atomic norm to JFS setting [35]. To develop a norm description of the JSS, we also extend the atomic norm to JSS setting and give the definition of CA-norm of JSS. The “*ℓ*_0_-norm” type atomic norm [38] is defined as

(28) $${\left\|r\right\|}_{A,0}=\inf\left\{s:r=\sum_{k=1}^{K}\sigma_{k}a(\xi_{k})\right\}$$

and it convex relaxation, the atomic norm [38], is defined as

(29) $${\left\|r\right\|}_{A}=\inf\left\{\sum_{k}\sigma_{k}:r=\sum_{k=1}^{K}\sigma_{k}a(\xi_{k})\right\}$$

where, *σ_k_* ≥ 0 as it is denoted as the power of signal.

The “*ℓ*_0_-norm” type CA-norm of signal from VULA is defined as

(30) $${\left\|R\right\|}_{CA,0}=\inf\left\{{\left\|z_{c}\right\|}_{A,0}+\sum_{j\in \Lambda }{\left\|z_{j}\right\|}_{A,0}:z_{c}+z_{j}=r_{j},j\in \Lambda \right\}$$

With the consideration of Equation (20), the goal of DOA estimation problem becomes the minimization of ‖*R*‖*_cA_*_,0_ satisfying the measurement a-priori

(31) $$\begin{array}{ccc} \underset{R}{\min}{\left\|R\right\|}_{CA,0} & s.t. & w_{j}=\Psi_{j}r_{j},j\in \Lambda \end{array}$$

We study the spark of continuous dictionary **Φ** in Equation (20). The quantity spark of **Φ**, denoted by spark(**Φ**), is the smallest number of atomic of **Φ** which are linearly dependent. spark(**Φ**) = spark(**Φ***_c_*) + Σ*_j_*_∈Λ_ spark(**Φ***_j_*) ∈ [2, *M* + 1]. Considered as a distributed model of [39], $r_{j}=\sum_{k=1}^{K}\sigma_{k}a_{j}(\xi_{k})$, *j* ∈ Λ is the unique optimizer to Equation (31) if

(32) $$K<\frac{\text{spark}(\Phi_{c})+\sum_{j\in \Lambda }\text{spark}(\Phi_{j})-1+\sum_{j\in \Lambda }\text{rank}(\omega_{j})}{2}$$

where, the atomic decomposition above ia the unique one satisfying that *K* = ‖***R***‖_
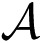
__,0_. It is the theoretical guarantees of the “*ℓ*_0_-norm” type CA norm minimization in Equation (31).

### 3.2. Joint SDP Based CA-Norm Minimization Algorithm

In this subsection, we describe how to use a joint SDP (JSDP) approach to solve the DOA estimation problem. The optimization problem in Equation (31) is computationally infeasible given the infinite dimensional formulation of the “*ℓ*_0_-norm” type CA norm in Equation (30). We attempt to provide a finite dimensional formulation in the following results. The goal of DOA estimation problem ‖***R***‖*_C 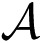
_*_,0_ in Equation (29) equals the optimal value of the following rank minimization problem [39]

(33) $$\begin{array}{ccc} \underset{u,n,U\geq 0}{\min}\text{rank}(U) & s.t. & U=\left[\begin{array}{cc} \text{d}-\text{toep}(u) & Z\\ Z^{*} & n \end{array}\right] \end{array}$$

where, $Z={\left[z_{c}^{*},z_{1}^{*};\ldots ,z_{J}^{*}\right]}^{*}$, d-toep(*u*) is the block diagonal matrix

$$\text{diag}(\text{toep}(u_{c}),\text{toep}(u_{1}),\ldots ,\text{toep}(u_{J}))$$

composed of toeplitz matrices generated from complex vectors *u* = {*u_c_*,***u**_j_*, *j* ∈ Λ}; toep(***u***) is a symmetric toeplitz matric generated by the vector ***u***; and ***U*** ≥ 0 means that ***U*** is positive semideninite. It follows that is equivalent to the following low rank matrix completion (LRMC) problem [40]

(34) $$\begin{array}{c} \underset{u,Z,n}{\min}\frac{1}{2N}\left(\text{rank}(\text{toep}(u_{c}))+\sum_{j\in \Lambda }\text{rank}(\text{toep}(u_{j})\right)\\ s.t.\left[\begin{array}{cc} \text{d}-\text{toep}(u) & Z\\ Z^{*} & n \end{array}\right]≽0,w_{j}=\Psi_{j}(z_{c}+z_{j}),j\in \Lambda \end{array}$$

Due to the NP-hard nature of rank minimization problem, solving the “*ℓ*_0_ norm” type CA-norm minimization would become computationally intractable. An alternative approach is to consider its convex relaxation, CA-norm, defined as

(35) $${\left\|R\right\|}_{CA}=\inf\left\{{\left\|z_{c}\right\|}_{A}+\sum_{j\in \Lambda }{\left\|z_{j}\right\|}_{A}:z_{c}+z_{j}=r_{j},j\in \Lambda \right\}$$

The atomic norm defined for single vector in Equation (29) is actually a special case of CA-norm for *J* = 1. And the similar study of total variance norm is introduced in [15]. In this work, we propose to solve the following CA-norm minimization problem to achieve accurate DOA estimations of the off-grid sparse spatial signals

(36) $$\begin{array}{cccc} \underset{R}{\min}{\left\|R\right\|}_{CA} & s.t. & w_{j}=\Psi_{j}x_{j}, & j\in \Lambda \end{array}$$

For ***r**_j_* = *z_c_* + *z_j_* ∈ ℂ*^N^*, *j* ∈ Λ,

(37) $${\left\|R\right\|}_{CA}=\inf\left\{\begin{array}{cc} \frac{1}{2N}\left(\text{tr}(\text{toep}(u_{c}))+\sum_{j\in \Lambda }\text{tr}(\text{toep}(u_{j}))\right)+\frac{1}{2}n: & \left[\begin{array}{cc} \text{d}-\text{toep}(u) & Z\\ Z^{*} & n \end{array}\right]≽0 \end{array}\right\}$$

which is proved in [35]. Therefore, Equation (36) can be expressed as the following computationally tractable SDP

(38) $$\begin{array}{c} \underset{u,Z,n}{\min}\frac{1}{2N}\left(\text{tr}(\text{toep}(u_{c}))+\sum_{j\in \Lambda }\text{tr}(\text{toep}(u_{j})\right)+\frac{1}{2}n\\ s.t.\left[\begin{array}{cc} \text{d}-\text{toep}(u) & Z\\ Z^{*} & n \end{array}\right]≽0,w_{i}=\Psi_{j}(z_{c}+z_{j}),j\in \Lambda \end{array}$$

We can get accurate ***Z*** by using SDP solver. The trigonometric polynomials about the DOAs information of common and innovation sources are given as

(39) $$p_{c,2N-2}(e^{-j2\pi {\hat{\xi }}_{c}})={\left|\sigma_{c}\right|}^{2}-{\left|\left(A_{c}^{*}z_{c})({\hat{\xi }}_{c}\right)\right|}^{2}={\left|\sigma_{c}\right|}^{2}-\sum_{i=-N-1}^{N-1}u_{c,i}e^{-j2\pi i{\hat{\xi }}_{c}},u_{c,i}=\sum_{n}z_{c,n}{\bar{z}}_{c,n-i}$$

(40) $$p_{j,2N-2}(e^{-j2\pi {\hat{\xi }}_{j}})={\left|\sigma_{j}\right|}^{2}-{\left|\left(A_{j}^{*}z_{j})({\hat{\xi }}_{j}\right)\right|}^{2}={\left|\sigma_{j}\right|}^{2}-\sum_{i=-N-1}^{N-1}u_{j,i}e^{-j2\pi i{\hat{\xi }}_{j}},u_{j,i}=\sum_{n}z_{j,n}{\bar{z}}_{j,n-i}$$

$p_{c,2N-2}(e^{-j2\pi {\hat{\xi }}_{c}})$ and $p_{j,2N-2}(e^{-j2\pi {\hat{\xi }}_{j}})$ have at most 2*n* − 2 roots, respectively [37]. Since construction $p_{2N-2}(e^{-j2\pi \hat{\xi }})$ is a real-valued and nonnegative trigonometric polynomial, it cannot have single roots on the unite circle since the existence of single roots would imply that $p_{2N-2}(e^{-j2\pi \hat{\xi }})$ takes on negative values. Therefore, $p_{2N-2}(e^{-j2\pi \hat{\xi }})$ is either zero everywhere or has at most *N* − 1 roots on the unit circle. The accurate DOAs of the sources can be estimated as

(41) $$\hat{\theta }=2\hat{\xi }-1$$

### 3.3. Dual Certificate

Dual problem is studied to check the successful reconstruction of the optimization [38]. Let R* denote the optimal solution to Equation (36) and $Q={\left[q_{1}^{*},\ldots ,q_{J}^{*}\right]}^{*}$, where *q_j_* ∈ ℂ*^Mj^*. Then the dual problem of Equation (36) is given as

(42) $$\begin{array}{ccc} \underset{Q}{\max}{\langle \Phi^{*}Q,R*\rangle }_{\mathbb{R}} & s.t. & {\left\|\Phi^{*}Q\right\|}_{CA}^{*}\leq 1 \end{array}$$

where ${\left\|\cdot \right\|}_{CA}^{*}$ is the dual norm of CA-norm, and

(43) $$\begin{array}{l} {\left\|\Phi^{*}Q\right\|}_{CA}^{*}=\underset{\left\|R\right\|C_{A=1}}{\sup}{\langle \Phi^{*}Q,R*\rangle }_{\mathbb{R}}\\ =\underset{{\left\|z_{c}\right\|}_{A}+{\sum_{j}\left\|z_{j}\right\|}_{A=1}}{\sup}\left({\langle \sum_{j\in \Lambda }\Phi_{j}^{*}q_{j},z_{c}\rangle }_{\mathbb{R}}+{\langle \sum_{j\in \Lambda }\Phi_{j}^{*}q_{j},z_{j}\rangle }_{\mathbb{R}}\right)\\ =\underset{\sigma_{c}+\sum_{j}\sigma_{j}=1,f_{c},f_{j}\in [0,1]}{\sup}\left(\sigma_{c}{\langle \sum_{j\in \Lambda }\Phi_{j}^{*}q_{j},a(f_{c})\rangle }_{\mathbb{R}}+{\langle \sum_{j\in \Lambda }\Phi_{j}^{*}q_{j},a(f_{j})\rangle }_{\mathbb{R}}\right)\\ =\underset{f\in [0,1]}{\sup}\max\left\{\left|\langle \sum_{j\in \Lambda }\Phi_{j}^{*}q_{i},a(f)\rangle |,\underset{j\in \Lambda }{\max}|\langle \Phi_{j}^{*}q_{i},a(f)\rangle \right|\right\} \end{array}$$

Strong duality simply holds since Equation (36) is only equality constrained and thus satisfies Slater's condition [27]. Based on this certification, a dual certificate to the optimality of the solution of Equation (36) can be obtained. And we proved its uniqueness [35] and provide it as a guide for the construction of dual polynomials.

## 4. Numerical Experiments

In this section, we evaluated the proposed approach by performing numerical experiments. We choose the probability of success as the major performance. The DOA estimation is considered as successful if the DOA estimation error of each source satisfies the following condition

(44) $$\max\left\{\underset{\xi_{c,k}\in \Omega_{c}}{\max}\{{\left\|{\hat{\xi }}_{c,k}-\xi_{c,k}\right\|}_{2}\},\underset{\xi_{j,k}\in \Omega_{j}}{\max}\{{\left\|{\hat{\xi }}_{j,k}-\xi_{j,k}\right\|}_{2}\}\right\}\leq 10^{-4}$$

We focus on the key impact factor of array aperture size and evaluate the demand of sensors in each SLA via different DOA estimation methods with multiple parameter settings. DOAs were generated uniformly random on [0,1] with an additional constraint on minimum separation Δ as follows

(45) $$\Delta =\underset{j}{\min}\underset{\xi ,\xi^{\prime }\in \Omega_{c}\cup \Omega_{j};\xi \neq \xi^{\prime }}{\inf}\left|\xi -\xi^{\prime }\right|\geq \frac{1}{N^{2}}$$

The inter-element spacings of SLAs were generated uniformly random on [λ/2, 40(λ/2)], which is integer multiple of the half wavelength. JSDP in Equation (38) was solved via SDPT3-4.0 toolbox [41].

**Figure 4.**
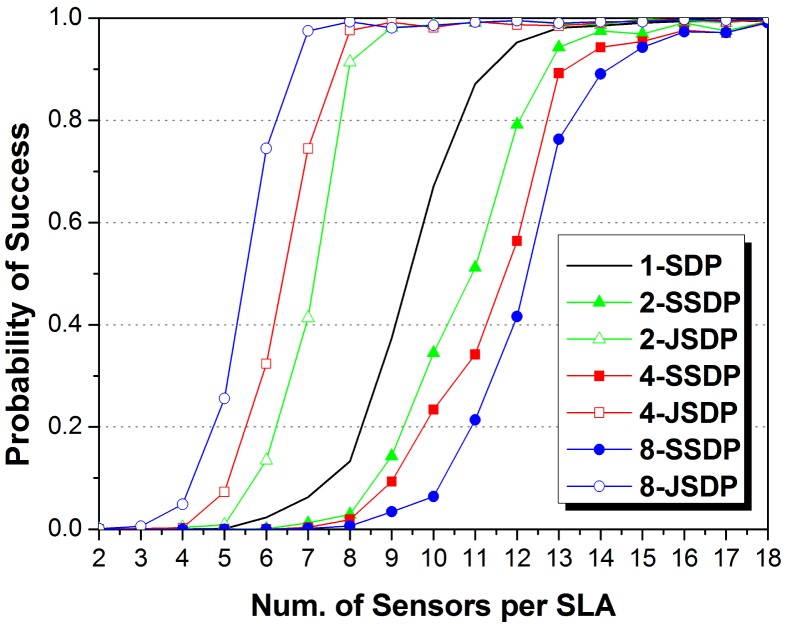
The impact of the number of sensors per SLA on probability of success DOA estimation with various numbers of SLAs compared between the joint SDP and separate one.

Firstly, we compare the proposed JSDP with the original separate-SDP (SSDP) approach in the ULAs. SSDP means joint calculation of the observed signals without any co-arrays methods. We set *K_c_* = 4, *K_j_* = 2 in each signal ensemble. We performed Monte Carlo experiments for *M_j_* from 2 to 18 and *J* = 1, 2, 4, 8, and recorded the probability of success from 2000 trials. The number of sensors in the VULA in JSDP is assumed as *N* = 40 to ensure that all the sources for SLAs in system can be localized. Figure 4 shows the performance curves.

The JSDP approach exhibits a definite advantage over its separate counterpart. In SSDP, one SLA requires at least 7 sensors to possibly localize 6 sources, and the increase of array number brings about larger calculation errors so as to decrease the probability of success. JSDP is equality to SSDP when *J* = 1, which requires approximately 11.7 sensors to successfully localize 6 sources with high probability. The JSDP approach achieves exact DOA estimation after *M_j_* exceeds a certain threshold, which is the demand of approximately 8.9 sensors in each SLA for *J* = 2, while at least 12.6 sensors per SLA are required for SSDP to achieve comparable performance. The gap increases with the increase of *J*, implying the promise of application to multi-arrays systems. When *J* = 4 and *J* = 8, JSDP respectively requires approximately 7.1 and 6.4 sensors to address the requirement of successful DOA estimations; SSDP respectively requires at least 15 sensors. Obliviously, JSDP makes the sensors in all the SLAs virtually construct a larger array to implement the DOA estimation of common sources which brings about larger degrees of freedom. In other words, we can use the arrays with smaller aperture size via JSDP to satisfy the same demand of sources localized via SSDP. The increase of *J* decreases the aperture requirement and system cost of SLAs.

**Figure 5.**
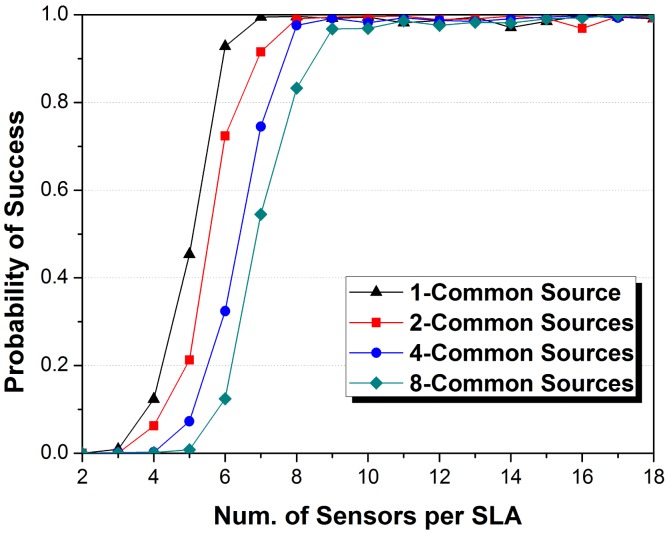
The impact of the number of sensors per SLA on probability of success DOA estimation with various numbers of common sources.

Then, we discuss the parameter impact of source number in detail. The number of common sources is the key object in the research closely related to the performance of JSDP. In the case of 4 SLAs system, 4 signal ensembles with 2 innovation sources are assumed. We performed Monte Carlo experiments for *M_j_* from 2 to 18 and the number of common sources *K_c_* = 1, 2, 4, 8, and recorded the probability of success from 2000 trials. The performance curves are shown in Figure 5.

When *J* = 1, approximately 6.2 sensors per SLA can successfully localize all 9 sources with high probability. As the increase of number of common sources, JSDP with original array aperture cannot obtain so many successful estimation and more sensors are required to allow more sources localized. In the situation with more localized sources, the sensors In short, an increasing number of common sources will increase the workloads of SLAs. The increase of *J* decreases the increment of sensors in each SLA. The total amount of sensors in the system is approximately proportional to the number of common sources according to the simulation results. We believe there has a relationship of the number of sensors and common sources. The theoretical bound will be studied in our next work.

**Figure 6.**
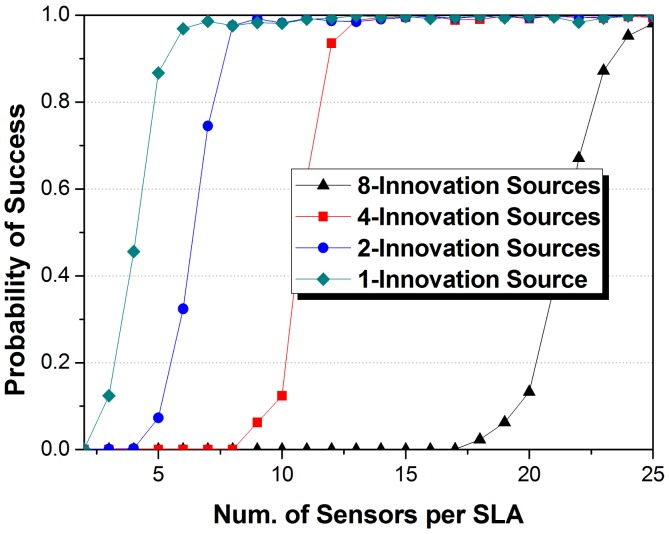
The impact of the number of sensors per SLA on probability of success DOA estimation with various numbers of innovation sources.

Of course, the impact of the number of innovation sources should also be studied to fully prove the high efficiency of JSDP. We discuss a SLAs system with 8 innovation sources in all, which means *J* × *K_j_* is a constant set as 8. Thus, *J* = 1, 2, 4, 8, corresponding to *K_j_* = 8, 4, 2, 1. And the number of common sources is still 4. We performed Monte Carlo experiments for *M_j_* from 2 to 25 and recorded the probability of success form 2000 trials. The number of sensors in the VULA is assumed as *N* = 80 to ensure that all the sources for SLAs in system can be localized. Figure 6 shows the performance curves.

As the average number of common and innovation sources per SLA is isocon descending with the number of SLAs, the demand of sensors per SLA is approximately proportional to the number of SLAs too. The demand of sensors when *J* = 1 is twice times as that when *J* = 2, and so on. The results fully indicate that all the sensors undertake the equal number of estimation task for common sources. On the other hand, the array aperture size is closely related to the number of sources in each SLA, which supports the definition of degree of freedom.

From the above simulation results, we can draw a conclusion that one source need about 2.3 sensors to estimate its DOA, which is similar to the conclusion in [37] to require at least two samples for a spike.

**Figure 7.**
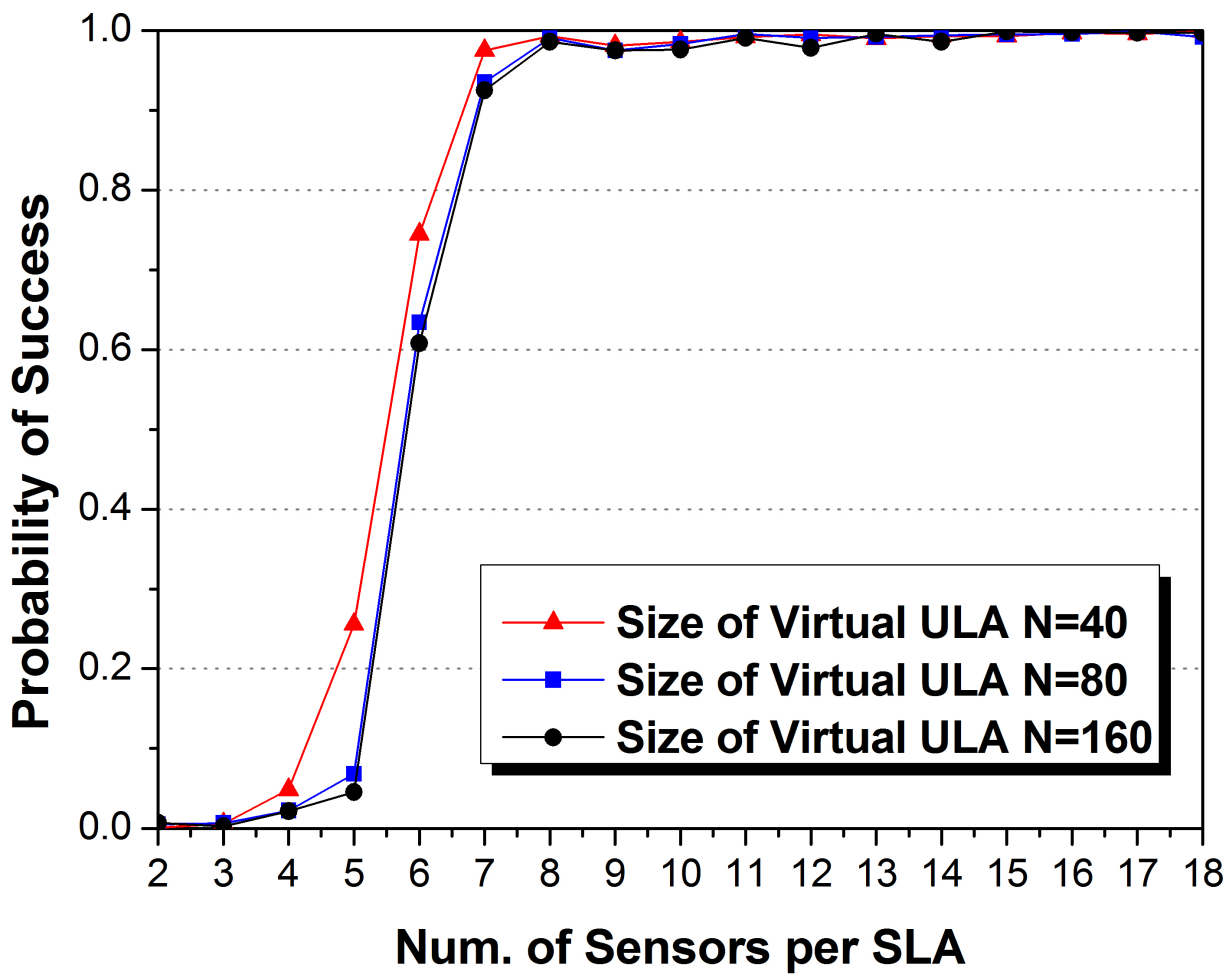
The impact of the number of sensors per SLA on probability of success DOA estimation with various numbers of sensors in the VULA.

Moreover, we also discuss the demand of sensors for the aperture size assumed in the VULA. We set *K_c_* = 4, *K_j_* = 2 in each ensemble. We performed Monte Carlo experiments for *M_j_* from 2 to 18 and *N* = 40, 80, 160, and recorded the probability of success from 2000 trials. The number of SLAs is set as *J* = 4. Figure 7 shows the performance curves.

Similar to all kind of DOA estimation methods, the aperture size of VULA has a threshold for enough degree of freedoms, in terms of the largest spacing difference of any two sensors in a SLA. If the array aperture addresses the requirement, the performance enhancement of algorithm becomes slow. Of courses, the ULA with larger aperture has higher degree of freedoms. However, the estimation accuracy is depended on the solution methods. In JSDP, the minimum resolution is determined by sensor numbers described in Equation (45). The finer resolution makes the DOA information in the case of two sources with a small DOA difference estimated more accurate so as to increase the probability of success.

## 5. Conclusions

The array aperture size is one of the most important parameters in array signal processing, highly effecting the accuracy of DOA estimations and the cost of location system. In this article, we discussed the application of multiple distributed SLAs with the coexistence of common and innovation sources, which was not referred to in the existing co-arrays methods. The separate algorithm for each SLA brings about the repeated calculations of DOA estimations for the common sources. The redundancy of the signal of common sources motivated us to address this problem with a joint DOA estimation method. All the sources for each SLA were classified into the common sources and the innovation ones. The common source was defined as the signal of which can imping on all SLAs in the system and the innovation one was defined as that can only imping on a specific SLA. Considering that the sources are sparse spatial distributed, we can use a JSS model to describe the problem of joint DOA estimation. We constructed a VULA for each SLA to obtain *O*(*N*) freedoms with *M_j_* sensors, and considered the signals observed by SLAs as the samplers of those observed by their corresponding VULAs. The relationship between these two kinds of signals is a random linear map. An off-grid synchronous approach was proposed to combine the observed signals in all the SLAs and jointly estimate the DOA information of all sources. A CA-norm was used to reformulate the problem of joint DOA estimation into the minimization problem, which was solved via JSDP. By exploiting the continuous counterpart of the JSS model, the DOAs of all sources can be simultaneously estimated with off-grid estimation accuracy. And the sources are localized with the arrays of smaller aperture in terms of the decrease of sensors via JSDP. From the numerical results, we can find the joint approach has more advantages than the existing separated one. All the estimation tasks are shared by all the SLAs which brings about at least 20% decrease of demand of sensors per SLA.

Of course, in this article we did the initial researches on the joint DOA estimation with distributed SLAs. Some more complex conditions such as the noise environment and different number of sensors per SLA (like co-prime arrays) should be considered in our next works. Although we conclusion the impacts of SLA number, source number and sensor number per VLUA on the demand of sensors in the SLAs, the formulation indicating the relationship between sensor number and the above parameters will be studied in detail.

## Abbreviation Name

CA-norm: Concatenated atomic norm
CS: Compressed sensing
DOA: Direction of arrival
DOF: Degree of freedom
DCS: Distributed compressed sensing
JFS: Joint frequency sparse
JSS: Joint space sparse
JSDP: Joint semidefinite programming
LRMC: Low rank matrix completion
MRLA: Minimization redundancy linear array
SDP: Semidefinite programming
SLA: Sparse linear array
SSDP: Seperate semidefinite programming
ULA: Uniform linear array
VULA: Virtual uniform linear array
